# Evaluating the Effect of Chinese Environmental Regulation on Corporate Sustainability Performance: The Mediating Role of Green Technology Innovation

**DOI:** 10.3390/ijerph19116882

**Published:** 2022-06-04

**Authors:** Min Wang, Youshi He, Jianya Zhou, Kai Ren

**Affiliations:** 1 School of Management, Jiangsu University, Zhenjiang 212013, China; wangmin2371@163.com (M.W.); zjy3045@163.com (J.Z.); 2 School of Finance & Economics, Jiangsu University, Zhenjiang 212013, China; kairen31@126.com

**Keywords:** environmental regulation, Porter hypothesis, green technology innovation, sustainability performance

## Abstract

The environmental pollution that accompanies economic growth has always been of widespread concern. The chemical industry is a highly energy-consuming industry in China, and the pollution this industry causes to the environment cannot be ignored. The paper is based on the Porter hypothesis and uses data from different regions of China. In this paper, we investigate the mediating role of different types of environmental regulation (divided into command-controlled, market-incentive, and voluntary environmental regulation) in positively affecting sustainability performance through green technology innovation (divided into green product innovation, green process innovation, and end-of-line management innovation). The results show that different versions of the Porter hypothesis can be accepted in Chinese chemical enterprises. This finding demonstrates that environmental regulation positively impacts both green technology innovation and sustainability performance. Green technology innovation plays a mediating role between environmental regulation and sustainability performance, especially in East China. However, the mediating effect of green product innovation is not significant. Further study shows that command-controlled environmental regulation has a more significant positive effect on sustainability performance. This suggests that the market-incentive and voluntary environmental regulation tools do not fully play their functional roles. Thus, the paper demonstrates the developmental shortcomings of environmental regulation, green technology innovation, and sustainability performance. This is more conducive to chemical enterprises improving green technology innovation and achieving long-term development and ecological environment protection.

## 1. Introduction

Balancing the environmental pollution problems arising from economical development has been a hot topic of research. Since the 1970s, China has enacted more than 1800 environmental protection-related policies to reduce pollutant emissions from industrial production [1]. Environmental regulation is a tool for government intervention in environmental resources and economic and social development. The government intervenes using administrative orders, regulations, and economic instruments. This approach improves the utilization of resources and ultimately leads to the redistribution of social wealth and the maximization of social welfare [2]. The main role of environmental regulation is to promote green technological innovation and to constrain the emission of pollutants during production [3]. Many issues of environmental regulation and environmental protection need to be explored from both theoretical and practical perspectives.

So far, environmental regulation has been used as an effective tool to combat environmental pollution, helping to curb the emission of pollution and achieve economic growth [4]. The effectiveness of environmental regulation is influenced by the level of the regional economy. The slower the economic development process of a region, the less effective the implementation of environmental regulations [5]. Zhang et al. [6] found that environmental regulation significantly reduced environmental pollution by analyzing provincial panel data for China from 2006 to 2017. The intensity of environmental regulation affects the effectiveness of environmental pollution, and only high-intensity environmental regulation can reduce pollution [7]. However, some scholars argue that environmental regulation does not inhibit environmental pollution. Using provincial panel data from 2004 to 2019, Li et al. [8] found that there is a “green paradox” in environmental regulation. From the perspective of the shadow economy, Zhang and Xu [9] found no relationship between environmental regulation and the reduction of environmental pollution. With the increase in the number of research perspectives, scholars began to study the relationship between informal environmental regulation with public participation and environmental pollution. It was found that public concern about environmental issues can influence the level of government environmental governance [10], indirectly reducing environmental pollution by companies.

Chemical enterprises, as heavy polluters, have high-intensity industrial activities that lead to serious excesses of pollutant emissions. This has caused a regional stress effect, leading to changes in ecosystem response mechanisms and an increase in the intensity of urban ecological risks [11]. With the application of the Porter hypothesis, environmental regulation has gradually become the main tool used to influence pollutant emissions in the chemical industry. Wang et al. [12] used chemical companies as a research sample and argued that environmental regulation can solve the pollution problem of heavily polluting companies. Moreover, environmental regulations can help chemical enterprises attract more investment [13] and improve their sustainability performance [14], ecological welfare performance [15], and the quality of environmental information disclosure [16]. Some scholars have also categorized environmental regulation for research. Guo and Jia [17] found that the influence of market-incentivized environmental regulation is significant in East China, while the influence of controlled environmental regulation is significant in central and West China. Therefore, when applying the Porter hypothesis to the chemical industry, it is important to reflect the differences between the chemical industry and other industries. In this paper, based on the platform of the Chinese chemical industry, the weak version, the strong version, and the narrow version of the Porter hypothesis were tested by analysis of the mediating effect. This not only lays a theoretical foundation for the deepening of environmental regulation in the Chinese chemical industry, but also provides a reference for the improvement of environmental regulation in other industries.

The paper extends the study of environmental regulation, green technology innovation, and innovation performance in a variety of ways. The contributions of this paper are as follows:

(a) This paper reveals the shortcomings of the sustainability performance of Chinese chemical companies and gives options for improvement. This improvement will modify the sustainable development direction of the business and promote sustainable performance. This development direction can also be used by other business owners, decision-makers, and managers.
(b) The impact of environmental regulation on firm innovation is complex [18]. Product innovation can lead to better-performing products. This may accelerate the obsolescence of old products and even change consumer behavior and reshape the market. Therefore, the impact of environmental regulation on product innovation in green technology innovation needs to be discussed. For this purpose, we classify both environmental regulation and green technological innovation into three types and analyze the interrelationship between them and sustainability performance, which has been neglected in previous studies.
(c) On the theoretical side, most scholars focused on the verification of the weak version of the Porter hypothesis. This paper examines the path of green development of chemical enterprises. We theoretically confirm the existence of different versions of the Porter hypothesis in Chinese chemical enterprises and enrich this theory.

The rest of this paper is organized as follows. Section 2 provides a review of the relevant literature and our hypotheses. Details of the analysis are given in Section 3, including data analysis and model tests. Section 4 shows the conclusion and policy recommendations. Section 5 presents research limitations and directions for future research.

## 2. Literature Review and Hypothesis

### 2.1. Environmental Regulation and Green Technology Innovation

Joseph Alois Schumpeter considers technological innovation as the integration of existing resources and equipment based on traditional technologies to create new technologies with social and economic benefits [19]. He argued that innovation is the main driver of socio-economic development and that only technological change is profitable. For ecological pollution and development, technological effects are the most studied elements. Hicksian (1932) was the first to introduce the concept of technology effects, arguing that changes in product prices enable firms to focus on innovation [20]. Several subsequent studies have found that prices do not significantly promote firm innovation [21]. Therefore, we can consider environmental regulations that restrict the production and emission of highly polluting raw chemical materials to be consistent with this situation [18].

In 1995, Michael Porter proposed the famous Porter hypothesis from a dynamic perspective [22]. He argued that well-designed environmental regulation policies can promote green technological innovation and that the costs disappear over time. Subsequently, Jaffe et al. [23] constructed a system of indicators to test the Porter hypothesis and generalized the Porter hypothesis into three versions. Scholars have since successively validated Jaffe’s view [24,25]. The weak version of the Porter hypothesis suggests that environmental regulations can lead to an increase in R&D investment and improve technological innovation. The strong version of the Porter hypothesis argues that well-designed environmental regulations can produce a significant innovation compensation effect and improve economic performance and competitiveness. The narrow version of the Porter hypothesis suggests that certain types of environmental regulations are more likely to stimulate firms to innovate, such as market-based environmental regulations. The main reason is that market-based environmental regulation has more flexibility in market instruments and has a significant informational advantage [26,27].

Scholars have different views on the types of environmental regulation. Some scholars divided environmental regulation into mandatory environmental policy and environmental economic policy [28], while others divided environmental regulation into social and economic types based on institutional arrangements [29]. Environmental regulation can also be divided into formal and informal environmental regulations depending on the implementation requirements [3]. As the research progresses, most scholars classify it into three types: command, market, and voluntary [30]. This is also the way this paper is classified. Various types of environmental regulations produce very different effects [31].

Finally, it is necessary to analyze the types of green technology innovation. There are differences between green product innovation and process innovation. Changes in production processes can encourage new product innovations [32]. However, technological innovation does not necessarily lead to product innovation [18]. This is the reason for the categorical discussion of green technology innovation in this paper. The earliest study in China was in 1998 when Yang Invention et al. classified green technology innovations into clean process innovation, end-of-pipe governance technology innovation, and green product innovation based on their functions [33]. The classification of later scholars was mostly based on this and varied according to the study area or sample [34,35,36]. This study divides green technology innovation into green product innovation, green process innovation, and end-of-line management innovation.

Thus, this paper proposes Hypothesis 1:

**Hypothesis** **1** **(H1).** *Different types of environmental regulations have a positive correlation with different types of green technology innovations.*

Command-controlled environmental regulation has characteristics such as being compulsory and authoritative, setting strict green technology innovation requirements and pollutant emission standards [34,37]. Command-controlled environmental regulation can raise the input standards of green products and technologies for enterprises and require green products to comply with domestic environmental certifications [38]. Command-controlled environmental regulation would set a red line for emissions [35]. The enterprises for which pollution emissions cross the red line are punished by forcing them to stop production; shut down; and rectify, revoke, and suspend relevant permits. Therefore, this paper proposes the next hypothesis.

**Hypothesis** **1-1.** *Command-controlled environmental regulation has a positive correlation with green product innovation* (H1-1a)*, green process innovation* (H1-1b)*, and end-of-line management innovation* (H1-1c).

Market-incentive environmental regulation covers all enterprises through market-based instruments to improve green technology innovation. This type of environmental regulation can compensate for R&D losses and offset some of the research costs [36]. Therefore, market-incentive environmental regulation can address the distorted allocation of social resources and correct the market failure of corporate green process innovation [39]. Based on the above analyses, we propose:

**Hypothesis** **1-2.** *Market-incentive environmental regulation has a positive correlation with green product innovation* (H1-2a)*, green process innovation* (H1-2b)*, and end-of-line management innovation* (H1-2c).

Voluntary environmental regulation is an informal form of environmental regulation with a low binding effect [40]. A company that is aware of eco-environmental protection is expected to consciously carry out green technological innovation and to strive to transform and upgrade its production methods. In the context of promoting sustainable development, companies that fail to innovate will be gradually eliminated in the market. This will result in some companies being pressured by market competition and public opinion [41]. An enterprise with a long-term pattern is supposed to maintain its reputation and sustain ecological sustainability through green technological innovation [42]. Therefore, this paper proposes the next hypothesis.

**Hypothesis** **1-3.** *Voluntary environmental regulation has a positive correlation with green product innovation* (H1-3a)*, green process innovation* (H1-3b)*, and end-of-line management innovation* (H1-3c).

### 2.2. Green Technology Innovation and Sustainability Performance

Sustainability performance consists of economic, environmental, and social performance [39] and is an important component of business development. The sustainable development of enterprises can be achieved through green technological innovation [43]. Formally, product innovation is the result of technological innovation. Environmentally friendly products help to reduce or avoid environmental burdens. Technological innovation involves process innovation, which ensures the standardization, simplicity, and efficiency of the production process. This can reduce production costs, increase productivity, generate process compensation, and ultimately affect corporate performance [44]. Compared with traditional product innovation, environmental product innovation helps to reduce or avoid environmental burdens. Thus, product innovation can combine cost and environmental benefits. Both process innovation and product innovation are variables that moderate the relationship between environmental and sustainability performance, but product innovation mediating variables play an important role [44]. Product innovation can also increase resource efficiency, increase sales returns, develop new markets, and improve the corporate image [45,46]. The impact of clean technology on sustainability performance cannot be ignored. Kerr and Newell [47] argue that the regulatory content is influencing the sustainability performance of firms through the use of clean technologies. Thus, the most controversial issue in this research area is the inconsistent relationship between environmental regulation and technological innovation based on different samples and research methods. This is also the research question of this study. Based on the above analyses, we propose:

**Hypothesis** **2.** *Green product innovation* (H2a)*, green process innovation* (H2b)*, and end-of-line management innovation* (H2c) *have a positive correlation with sustainability performance.*

### 2.3. Environmental Regulation and Sustainability Performance

The version of the strong Porter hypothesis suggests that environmental regulation not only promotes enterprises’ innovation, but also enhances competitiveness or performance by compensating for the costs of innovation [48]. Scholars who support this version argue that the strength of environmental regulation can largely improve enterprises’ sustainability performance through green technology innovation [49,50]. Thus, when the government implements strict environmental regulation policies on enterprises, it has an impact on sustainability performance by promoting green technology innovation [51]. Moreover, the increase in sustainability performance is mainly characterized by technological progress, which can reduce energy efficiency between “energy/labor” and is expressed as an energy rebound effect [52]. Thus, environmental regulation can promote the productivity of firms, which is a key factor for their sustainable development [35].

Environmental regulation requires companies to disclose the implementation of their environmental and social strategies, which can enhance their sustainability performance [39]. Monitoring the implementation of strategies by enterprises is an element of both command-controlled and voluntary environmental regulation.

Environmental regulation can also influence green sustainability performance by affecting enterprises’ finances [42]. Market regulation approaches such as credit incentives and lower taxes by the government can reduce the financial pressure on enterprises. This allows the company to remain up and running and in a good financial condition while maintaining green technological innovation, resulting in a “cost compensation effect” [53]. Therefore, this paper proposes the following hypothesis:

**Hypothesis** **3.** *Command-controlled environmental regulation* (H3a)*, market-incentive environmental regulation* (H3b)*, and voluntary environmental regulation* (H3c) *have a positive correlation with sustainability performance.*

**Figure 1:**
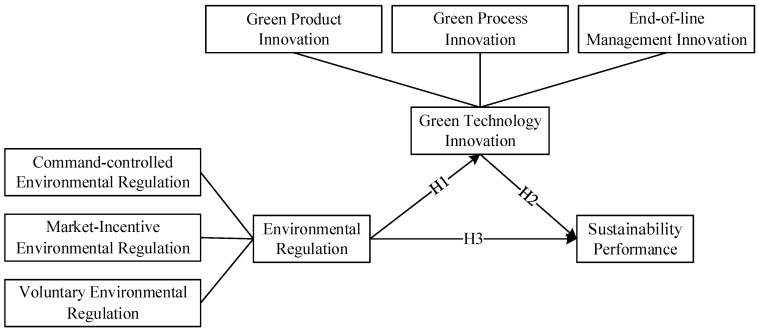
Research design.

The technical roadmap of this paper is as follows (Figure 1):

## 3. Methodology

### 3.1. Sample and Population

We selected one chemical firm in each county of China to obtain valid data. The survey began on 1 September 2020 and ended on 30 March 2021 (Appendix A). A total of 1000 questionnaires were distributed, and 766 questionnaires were returned, with a return rate of 76.6%. After eliminating 205 invalid questionnaires or questionnaires of low quality, 561 questionnaires were left. There are geographical differences in the chemical output value in China. On the premise of matching the output value of chemical enterprises with the sample size, 400 samples were selected. The survey method was a mainly online questionnaire, supplemented by interviews, commissioned interviews, and mail. The sample characteristics are shown in Table 1.

### 3.2. Measures

After reviewing references on environmental regulation [54,55,56], green technology innovation [55,57,58], and sustainability performance [59,60], we constructed indicators for measurement and then translated the indicators into survey questions.

The environmental regulation questionnaire consisted of command-controlled environmental regulation (M = 4.58, SD = 0.46), market-incentive environmental regulation (M = 4.04, SD = 0.39), and voluntary environmental regulation (M = 3.93, SD = 0.40).

The questionnaire for green technology innovation consisted of green product innovation (M = 3.11, SD = 0.37), green process innovation (M = 3.48, SD = 0.40), and end-of-line technology governance capabilities (M = 3.77, SD = 0.43).

The questionnaire on chemical companies’ sustainability performance (M = 3.09, SD = 0.37) was constructed in terms of both economic and environmental benefits.

Data were collected through questionnaires using a 7-point scale ranging from 1 = not at all to 7 = to a great extent.

### 3.3. Exploratory Factor Analysis

Exploratory Factor Analysis (EFA) is a valid method to test the credibility of the data to determine if the measurements are consistent. It is recommended that the ratio of the number of items to the number of cases should be greater than 1:10 [61], so this sample size (N = 400) was adequate for EFA. The sample adequacy was also assessed by the Kaiser_Mayer_Olkin (KMO) measure of sampling adequacy. KMO should be no less than 0.5 (environmental regulation KMO = 0.888, green technology innovation KMO = 0.820, and green sustainability performance KMO = 0.917) [62].

The internal consistency values were assessed using Cronbach’s alpha [63]. It was determined that if the Cronbach’s alpha reached 0.7 or above [64], the tool would be deemed to have internal consistency. Finally, we tested composite reliability to determine whether the items are consistent with each other and are reliable. The results confirmed that all the items are authentic, as the value of construct was higher than the cutoff value of 0.70 [64]. As shown in Table 2, our data are credible.

### 3.4. Confirmatory Factor Analysis

We excluded data that did not pass the reliability test and performed a validated factor analysis on the data using SPSS 24.0 [65,66]. The measurement models chosen for the paper were environmental regulation, green technology innovation, and green sustainability performance. As shown in Table 3, we found an acceptable model fit in terms of chisq/df, as the values of 1.72, 1.68, and 0.98 are less than 2 [67]. The other indicators, such as GFI, CFI, and NNFI, provided acceptable values (e.g., closed or above 0.90) [60]. Similarly, RMSEA also gave an acceptable value (e.g., below 0.080) [68].

### 3.5. Structural Models

In this structural model, we tested the hypotheses. We divided the sample into East China, Central China, and West China, according to the level of economic development (see Table 4) [69]. The results show that the model fit and the values (see Table 5) were found to be in the acceptable range [67].

**Figure 2:**
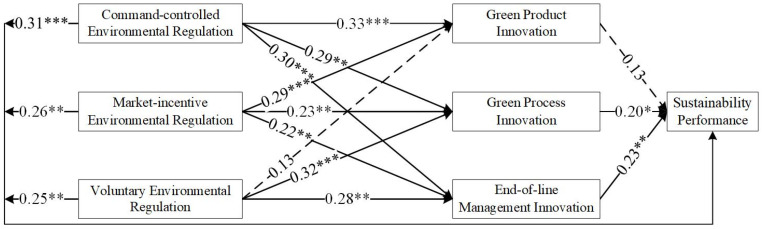
Results of the hypothesized model in China. * denotes *p* < 0.05; ** denotes *p* < 0.01; *** denotes *p* < 0.001.

**Figure 3:**
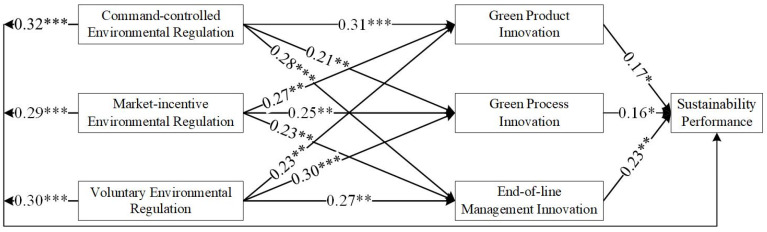
Results of the hypothesized model in East China. * denotes *p* < 0.05; ** denotes *p* < 0.01; *** denotes *p* < 0.001.

**Figure 4:**
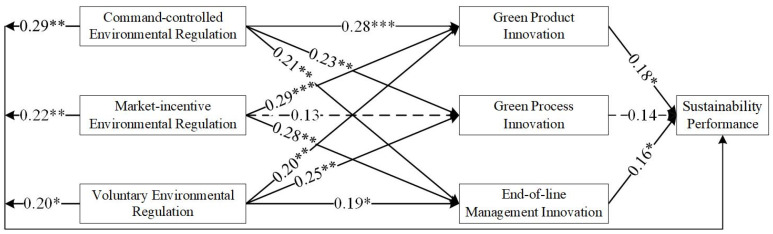
Results of the hypothesized model in Central China. * denotes *p* < 0.05; ** denotes *p* < 0.01; *** denotes *p* < 0.001.

**Figure 5:**
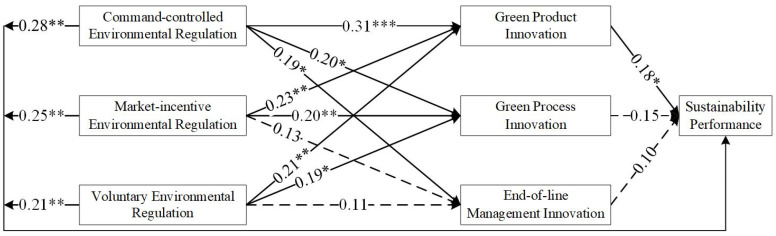
Results of the hypothesized model in West China. * denotes *p* < 0.05; ** denotes *p* < 0.01; *** denotes *p* < 0.001. In this study, the self-help sampling method (bootstrap method) was used to further test for mediating effects (N = 400), and Bias correction was used to detect confidence intervals (see Table 6).

As shown in Table 4, most of the results supported the hypothesis, although some of the results did not. Figure 2, Figure 3, Figure 4 and Figure 5 show the hypothesis test results, and the dashed line indicates that the results did not support the hypothesis.

### 3.6. Results

The test results for East China, Central China, and West China differed. H1 tested whether environmental regulations are positively associated with green technology innovation (H1-1, H1-2, and H1-3). As shown in Table 4 and Figure 2, based on the sample of China, the results support H1-1, H1-2, H1-3b, and H1-3c. H1-3a was not supported, implying a low willingness of firms to voluntarily participate in green technology innovation. In East China, the results supported all hypotheses. This may be related to the economic development of the eastern region. As the economic level increases, environmental regulation shows a ‘U’ relationship, which first constrains and then promotes technological innovation (e.g., [70]). For East China, the ‘U’ inflection point has been passed. In Central China, the results only did not support H1-2b. This indicates a gap in market incentives in the Central region. The results show that in the Western region, H1-2c and H1-3c were not supported. The economic level in West China is low, and the market incentives adopted are not working sufficiently well. Moreover, the willingness of companies to deal with pollutants on their own initiative is low. This means that in Central and West China, the impact has not yet passed the ‘U’ inflection point.

H2 tested whether green technology innovation is positively associated with sustainability performance (H2a, H2b, and H2c). As shown in Table 4 and Figure 2, based on the sample of China, the effect of green product innovation on sustainable development performance is not significant. However, all the hypotheses passed the test when the sample of East China was used. The direct relationship between green technology innovation and performance is more evident in East China as a developed region (e.g., [71]). This result is supportive of the RBA theory that firms with unique resources and capabilities can achieve a sustainable competitive position in the market [72]. In West China, H2b and H2c were not supported.

H3 tested whether environmental regulations are positively associated with sustainability performance (H3a, H3b, and H3c). As shown in Table 4 and Figure 2, Figure 3, Figure 4 and Figure 5, all tests passed. This is in line with the findings of numerous scholars (e.g., [57]).

In testing the mediating effect, the paper did not use a sample of regions. The paper tested the mediation effect with the total Chinese chemical firms as the sample. The indirect effect of environmental regulation on sustainability performance, via green technology innovation, was significant.

## 4. Conclusions and Discussion

Based on the results, the main conclusions of the paper are as follows:

(a) Environmental regulations can significantly improve the sustainability performance of chemical firms, which implies that the strong version of Porter hypothesis is acceptable in Chinese chemical firms. Therefore, the improvement of environmental regulation can facilitate the transformation and upgrading of chemical firms to green production and help them to obtain higher profits.
(b) Command-controlled environmental regulation has the most positive effect on sustainability performance. However, market-incentivized environmental regulations can be flexibly adapted to changes in the market and have greater potential for promoting green development in chemical companies [73]. Therefore, market-incentive environmental regulations can be used to reduce chemical enterprises’ resistance to environmental regulations.
(c) Environmental regulations can improve green technology innovation in Chinese chemical firms, which suggests that a weak version of the Porter hypothesis can be accepted by the Chinese chemical industry. However, some hypotheses are not supported. This indicates that the effect is not highly significant.
(d) Green technology innovation can improve the sustainability performance of chemical companies, but the impact of green product innovation is not significant. Therefore, chemical companies ignore product innovation or do not pay attention to green product innovation, which is an area for improvement.
(e) There is a mediating role of green technology innovation in environmental regulation and sustainability development performance. However, this mediating effect is not very significant. This requires the joint efforts of the government and chemical companies in order to target the formulation of environmental regulations needs and correct the direction of green technology innovation.
(f) There are disparities in the level of environmental regulation and green technology innovation in different regions of China. This may be related to factors such as the level of economic development and energy use efficiency [74].

Combining theory and conclusion, the paper argues that the deepening of the green development of Chinese chemical enterprises contains three directions. First, different policies are implemented for different types of environmental regulations. Second, the development of green technological innovation capabilities needs to be directional. Third, the incentives for sustainability performance need to be comprehensive. In this regard, the paper proposes the following policy recommendations.

The focus of command-controlled environmental regulation is on the green production chain. This chain requires the production process of chemical products to maintain low consumption and pollution and regulates the use of green process technologies. The standards for the use of pollution control technologies need to be echoed by the chemical industry’s emission standards. The monitoring of environmental pollution in the chemical industry ought to be increased, and stratified and batch treatment should be implemented for chemical enterprises with different pollution levels. Chemical enterprises with serious excess emissions of environmental pollution levels can be eliminated. The focus of market-based environmental regulation is to improve tradable emission permits and environmental tax. This requires limiting the purchase of tradable emission permits to avoid excessive purchases by chemical companies to avoid pollutant treatment.

Chemical companies need to pay attention to green product innovation. This includes strict control over the selection of raw materials for green products, product packaging, and sales, as well as the continuous research and development of green products. In the production process, the obsolete traditional processes of chemical companies are characterized by high energy consumption and pollution. Chemical enterprises should decisively eliminate the obsolete traditional processes or transform and upgrade the technology based on these processes. In terms of waste treatment technology, chemical enterprises can innovate and develop their technology. The use of a green supply chain has a positive effect on the sustainable development performance of enterprises [75]. Enterprises should improve their green supply chain system to reduce waste emissions and achieve low-carbon development.

Chemical companies improve their sustainability performance by improving their green management system. Conceptually, green management needs to integrate the awareness of environmental protection into the operation and management of chemical enterprises. This requires chemical enterprises to fully consider environmental protection requirements in their operation and management. At the same time, the traditional management thinking and mode of management should be improved, and the tendency toward the one-sided pursuit of profit while ignoring environmental resources and pollution should be changed. This can be considered in terms of green corporate culture, the development of green management strategies, the appropriate introduction of green investments, and the establishment of green functions.

## 5. Limitations and Future Research

In reality, the government will implement different environmental regulation tools in a targeted manner according to different regions’ economic development and environmental pollution levels. This can have certain cross-effects, which this paper does not take into account. In addition, the paper does not analyze the intensity of environmental regulations and the degree of technological progress in different economic regions. In the future, structural equation models can be constructed for different economic regions to investigate whether factors such as economic development level and pollution level affect the impact of environmental regulations on enterprises’ sustainability performance.

## Figures and Tables

**Table 1:** Sample characteristics.

Attribute	Categories	Number of Samples	Proportion (%)	Attribute	Categories	Number of Samples	Proportion (%)
Area	East China	150	37.5	Profit (RMB)	≤20 M	72	18
	Central China	136	34		20~40 M	80	20
	West China	114	28.5		40~60 M	136	34
Enterprise age	≤5 year	88	22		60~80 M	62	15.5
	6~10 year	126	31.5		80~100 M	32	8
	11~15 year	112	28		≥100 M	18	4.5
	16~20 year	56	14	Technical Staff (n)	≤20	36	9
	≥20 year	18	4.5		20~40	112	28
Enterprise type	State-owned	186	46.5		40~60	144	36
	Private	176	44		60~80	44	11
	Foreign	22	5.5		80~100	38	9.5
	Joint venture	16	4		≥100	26	6.5

**Table 2:** Factor loadings and Cronbach α.

	Factor Loadings	Cronbach α
Command-controlled environmental regulation	0.821	0.876
Market-incentive environmental regulation	0.722	0.891
Voluntary environmental regulation	0.729	0.800
Green product innovation	0.780	0.822
Green process innovation	0.719	0.818
End-of-line management innovation	0.800	0.806
Sustainability performance	0.718	0.826

**Table 3:** Results of model fitting.

	chisq/df	GFI	RMSEA	CFI	NNFI
Environmental Regulation	1.72	0.912	0.031	0.931	0.906
Green Technology Innovation	1.68	0.940	0.071	0.958	0.915
Green Sustainability performance	0.98	0.971	0.078	0.930	0.955

**Table 4:** Results of hypothesis testing.

Hypothesis	China			East China			Central China			West China		
	Std	t Value	Results	Std	t Value	Results	Std	t Value	Results	Std	t Value	Results
H1-1a	0.33 ***	7.81	support	0.31 ***	6.49	support	0.28 ***	4.33	support	0.31 ***	6.09	support
H1-1b	0.29 **	5.00		0.21 **	4.10		0.23 **	3.83		0.20 *	3.58	
H1-1c	0.30 ***	6.01		0.28 ***	4.66		0.21 **	3.50		0.19 *	3.00	
H1-2a	0.29 **	4.70		0.27 **	4.38		0.29 ***	4.88		0.23 **	3.91	
H1-2b	0.23 **	3.83		0.25 **	3.88		0.13	1.01	not support	0.20 **	3.49	
H1-2c	0.22 **	3.48		0.23 **	3.47		0.28 **	4.30	support	0.13	1.49	not support
H1-3a	0.13	1.57	not support	0.23 **	3.28		0.20 **	5.30		0.21 **	3.66	support
H1-3b	0.32 ***	7.62	support	0.30 ***	6.30		0.25 **	3.99		0.19 *	3.11	
H1-3c	0.28 **	4.65		0.27 **	4.18		0.19 *	2.78		0.11	1.38	not support
H2a	0.13	1.48	not support	0.17 *	3.08		0.18 *	2.96		0.18 *	2.90	support
H2b	0.20 **	2.89	support	0.16 *	2.91		0.14	1.91	not support	0.15	1.55	
H2c	0.23 **	3.09		0.23 **	3.88		0.16 *	2.68	support	0.10	1.27	not support
H3a	0.31 ***	6.78		0.32 ***	7.79		0.29 **	4.68		0.28 **	4.25	support
H3b	0.26 **	4.22		0.29 ***	5.17		0.22 **	3.28		0.25 **	4.05	
H3c	0.25 **	4.05		0.30 ***	6.29		0.20 *	3.01		0.21 **	3.77	

Note: * denotes *p* < 0.05; ** denotes *p* < 0.01; *** denotes *p* < 0.001, Std is the Standardized path coefficient.

**Table 5:** Model fitting results for different regions.

	chisq/df	GFI	AGFI	RMSEA	NFI	TLI	CFI
China	1.90	0.978	0.932	0.041	0.922	0.980	0.905
East China	0.127	0.955	0.972	0.038	0.971	0.925	0.930
Central China	0.092	0.933	0.979	0.040	0.988	0.927	0.946
West China	1.07	0.925	0.909	0.032	0.938	0.926	0.985

**Table 6:** Indirect effects of environmental regulations on sustainability performance through green technology innovation.

	Mediators	Indirect Effects	Bias Corrected 95% CI
CER → SP	Green product innovation	0.031	0.020 to 0.588
	Green process innovation	0.104	0.054 to 0.830
	End-of-line management innovation	0.098	0.019 to 0.725
MER → SP	Green product innovation	0.051	0.025 to 0.610
	Green process innovation	0.036	0.028 to 0.526
	End-of-line management innovation	0.025	0.016 to 0.313
VER → SP	Green product innovation	0.019	0.012 to 0.406
	Green process innovation	0.073	0.045 to 0.739
	End-of-line management innovation	0.038	0.030 to 0.594

Note: CER = Command-controlled environmental regulation, SP = Sustainability performance, MER = Market-incentive environmental regulation, VER = Voluntary environmental regulation.

## Appendix A

**Table A1:** Basic information.

Company Name						
Area:	a. East China	b. Central China	c. West China			
Enterprise type	a. State-owned	b. Private	c. Foreign	d. Joint venture		
Enterprise ag	a. ≤5 year	b. 6~10 year	c. 11~15 year	d. 16~20 year	e. ≥20 year	
Annual profit (RMB)	a. ≤20 M	b. 20~40 M	c. 40~60 M	d. 60~80 M	e. 80~100 M	f. ≥100 M
Technical staff (n)	a. ≤20	b. 20~40	c. 40~60	d. 60~80	e. 80~100	f. ≥100

**Table A2:** Questionnaire for command-controlled environmental regulation.

Item or Indicator	Inconformity ←--------→ Conformity						
	1	2	3	4	5	6	7
The government strictly regulates chemical companies to comply with exhaust emission standards.	□	□	□	□	□	□	□
The government strictly regulates chemical companies to comply with wastewater discharge standards.	□	□	□	□	□	□	□
The government strictly regulates chemical companies to comply with chemical solid waste discharge standards.	□	□	□	□	□	□	□
The government strictly regulates chemical companies to comply with standards for the use of green technology.	□	□	□	□	□	□	□
The government enforces very severe penalties on chemical companies that exceed emissions standards.	□	□	□	□	□	□	□

**Table A3:** Questionnaire for market-incentive environmental regulation.

Item or Indicator	Inconformity ←--------→ Conformity						
	1	2	3	4	5	6	7
The Government strictly regulates the sewage charges levied on chemical companies.	□	□	□	□	□	□	□
The government imposes a reasonable and regulated environmental tax on chemical companies.	□	□	□	□	□	□	□
The tradable emission permits issued by the government to chemical companies are in line with local development requirements.	□	□	□	□	□	□	□
The government sets up a reasonable and regulated special fund for technological progress.	□	□	□	□	□	□	□
The government offers reasonable and regulated credit concessions to chemical companies.	□	□	□	□	□	□	□

**Table A4:** Questionnaire for voluntary environmental regulation.

Item or Indicator	Inconformity ←--------→ Conformity						
	1	2	3	4	5	6	7
Voluntary participation agreements are regularly signed between the government and chemical companies.	□	□	□	□	□	□	□
The government is proactive in improving market regulation mechanisms for voluntary environmental regulation.	□	□	□	□	□	□	□
The government is proactive in disclosing information to the community about the environmental protection of chemical companies.	□	□	□	□	□	□	□
The government proactively monitors the voluntary participation of chemical companies.	□	□	□	□	□	□	□
The government is proactive in educating chemical companies on eco-ethical awareness.	□	□	□	□	□	□	□

**Table A5:** Questionnaire for green product innovation.

Item or Indicator	Inconformity ←--------→ Conformity						
	1	2	3	4	5	6	7
Chemical companies strictly regulate the choice of raw materials for green products.	□	□	□	□	□	□	□
The development of green product projects in chemical companies is ongoing.	□	□	□	□	□	□	□
Chemical companies develop green products that meet domestic environmental standards.	□	□	□	□	□	□	□
Green products developed by chemical companies are very easy to pass the domestic green product certification.	□	□	□	□	□	□	□
Chemical companies develop green products that are beneficial to the health of consumers.	□	□	□	□	□	□	□

**Table A6:** Questionnaire for green process innovation.

Item or Indicator	Inconformity ←--------→ Conformity						
	1	2	3	4	5	6	7
Emissions of industrial “three wastes” from chemical companies were significantly reduced.	□	□	□	□	□	□	□
Chemical companies have taken the initiative to upgrade their green process technologies.	□	□	□	□	□	□	□
The production process for green processes in chemical companies is clear and standardized.	□	□	□	□	□	□	□
Chemical companies are decisively phasing out obsolete processes that are heavy polluters.	□	□	□	□	□	□	□
Chemical companies are proactive in adapting traditional processes.	□	□	□	□	□	□	□

**Table A7:** Questionnaire for end-of-line management innovation.

Item or Indicator	Inconformity ←--------→ Conformity						
	1	2	3	4	5	6	7
Wastewater treatment technology for chemical companies is constantly being upgraded.	□	□	□	□	□	□	□
Waste gas treatment technology for chemical companies is constantly being upgraded.	□	□	□	□	□	□	□
Waste residue treatment technologies for chemical companies are constantly being upgraded.	□	□	□	□	□	□	□
Chemical companies are constantly upgrading their pollutant management equipment.	□	□	□	□	□	□	□
Research into the “three waste” treatment technologies for chemical companies is ongoing.	□	□	□	□	□	□	□

**Table A8:** Questionnaire for sustainability performance.

Item or Indicator	Inconformity ←--------→ Conformity						
	1	2	3	4	5	6	7
The actual return on a company’s green product is in line with the company’s expected return.	□	□	□	□	□	□	□
Green technology innovation keeps companies in good financial shape.	□	□	□	□	□	□	□
The ability of companies to innovate with green technology has increased significantly.	□	□	□	□	□	□	□
The cost of pollution control for companies has been significantly reduced.	□	□	□	□	□	□	□
The market share of a company’s green products is higher than that of its competitors in the market.	□	□	□	□	□	□	□
